# Serum inhibitory and bactericidal titers in the clinical management of bacterial infections

**DOI:** 10.1186/1471-2334-14-S7-O20

**Published:** 2014-10-15

**Authors:** Ioana Berciu, Oana Săndulescu, Anca Streinu-Cercel, Adrian Streinu-Cercel

**Affiliations:** 1 Carol Davila University of Medicine and Pharmacy, Bucharest, Romania; 2 National Institute for Infectious Diseases "Prof. Dr. Matei Balş", Bucharest, Romania

## Background

The clinical management of bacterial infection computes factors related to bacteria (e.g., resistance profile [1]), antibiotics (e.g., activity spectrum, distribution volume, etc.), host characteristics (e.g., vascularization of the infected tissue, effectiveness of host defenses, etc.), as well as pharmacokinetic (PK) parameters [2]. The serum inhibitory (SIT) and bactericidal titers (SBT) are laboratory tests that simulate the interactions between antibiotics and bacteria in the human body milieu.

## Methods

In the Emergency Laboratory of the Adults 2 Clinical Ward of the National Institute for Infectious Diseases "Prof. Dr. Matei Balş", we consistently perform SIT and SBT in complicated cases of bacterial infections. We have retrospectively collected and analyzed data from such tests performed between 2012 and 2014 and correlated the results with the patient’s overall clinical evolution. We present the descriptive results.

## Results

We have analyzed 89 cases. The serum antibiotics tested were: linezolid (15 cases, 16.9%), carbapenems (12 cases, 13.5%) with (5 cases) and without (7 cases) colistin, aminopenicillins (10 cases, 11.2%), and in smaller percentages: oxacillin, tigecycline, fluoroquinolones, trimethoprim/sulfamethoxazole, glycopeptides, aminoglycosides, ceftaroline and other cephalosporins.

In most cases serum samples were collected at the time of peak PK, with the exception of three cases where the trough concentration was examined instead. The tested germs were mostly *Staphylococcus* spp. (51.7%), followed by *Enterococcus* spp. (14.6%), *Streptococcus* spp. (9%) and Gram-negative bacilli (24.7%) such as: *E. coli*, *Klebsiella* spp. and *Pseudomonas* spp. The strains had been isolated from blood cultures (49.4%), cutaneous wounds (23.6%), tracheal aspirate (13.5%) and urine samples (7.9%).

SIT ranged from 1/2 (12.4%) to 1/512 (5.6%), but only in 20 cases (22.4%) the titers were in the therapeutic comfort range of 1/64 to 1/512. SBT ranged from 0 (11.2%) to 1/256 (6.7%). SIT and SBT were equal and in the comfort range for 2 strains of wound *Staphylococcus*, 4 strains of blood culture *Staphylococcus*, and 3 strains of tracheal aspirate *E. coli*.

## Conclusion

Interdisciplinary patient management and good collaboration with the bacteriology laboratory all contribute to establishing, maintaining and adapting targeted antimicrobial therapy.
